# Microbiome in urologic neoplasms: focusing on tumor immunity

**DOI:** 10.3389/fimmu.2024.1507355

**Published:** 2024-12-05

**Authors:** Jun Zou, Baisheng Xu, Hongbing Gao, Peiyue Luo, Tao Chen, Huanglin Duan

**Affiliations:** ^1^ Department of Otorhinolaryngology, The Affiliated Fengcheng Hospital of Yichun University, Fengcheng, Jiangxi, China; ^2^ Department of Urology, The First People's Hospital of Xiushui, Jiujiang, Jiangxi, China; ^3^ The First Clinical College, Gannan Medical University, Ganzhou, Jiangxi, China

**Keywords:** microbiome, urologic neoplasms, tumor immunity, immunotherapy, inflammation

## Abstract

Urological tumors are an important disease affecting global human health, and their pathogenesis and treatment have been the focus of medical research. With the in - depth study of microbiomics, the role of the microbiome in urological tumors has gradually attracted attention. However, the current research on tumor - associated microorganisms mostly focuses on one type or one site, and currently, there is a lack of attention to the microbiome in the immunity and immunotherapy of urological tumors. Therefore, in this paper, we systematically review the distribution characteristics of the microbiome (including microorganisms in the gut, urine, and tumor tissues) in urologic tumors, the relationship with disease prognosis, and the potential mechanisms of microbial roles in immunotherapy. In particular, we focus on the molecular mechanisms by which the microbiome at different sites influences tumor immunity through multiple “messengers” and pathways. We aim to further deepen the understanding of microbiome mechanisms in urologic tumors, and also point out the direction for the future development of immunotherapy for urologic tumors.

## 1 Introduction

Urologic tumors commonly include prostate cancer, bladder cancer, and kidney cancer. They not only pose a great threat to the life and health of patients but also put a heavy burden on the global healthcare system. Epidemiological data surveys show that in 2020, the global incidence of prostate cancer was 1,414,259, that of bladder cancer was 573,278, and that of renal cancer was 431,288, resulting in 375,304, 212,536, and 179,368 deaths respectively (1, 2). Compared with the previous year, the incidence rates of the three diseases have increased significantly (3). In addition, all three diseases showed an increasing incidence with age, which was especially evident above 50 years of age. In addition, they also exhibit significant gender differences (3, 4). These figures present a dismal situation. Moreover, with the trend of population aging and economic development, the incidence of these diseases will continue to increase, and the resulting burden will also increase (5). The occurrence of urologic tumors is associated with a variety of factors, including but not limited to genetics, environment, and lifestyle habits. To date, the complex interactions of these factors have led to a pathogenesis of urological tumors that has not been fully elucidated. As medical research progresses, our understanding of these tumors has improved. For example, prostate cancer may be associated with obesity (6), fitness (7), diabetes mellitus (8), dietary patterns (9), and vitamin E supplements (10); bladder cancer may be associated with smoking (11, 12), parasitic infections, and chronic inflammation (13); and renal cancer may be closely related to obesity (14), fruit or vegetable intake (15, 16), smoking (17), hypertension (17–19), and other factors. Treatment methods for urological tumors mainly include surgery, radiotherapy, and chemotherapy. Due to the complexity and diversity of urological tumors, the therapeutic efficacy often varies from person to person. In addition, the high recurrence rate of urological tumors is also a major problem in current treatment. Therefore, finding more effective treatment methods to improve the survival rate and quality of life of patients has become an important topic in the current research field of urological tumors. With the rapid development of biomedical technology, our understanding of urological tumors has penetrated to the molecular level. This enables us to diagnose the disease more accurately, assess the prognosis and develop personalized treatment plans. At the same time, the development and clinical application of novel drugs have also brought new hope for the treatment of urological tumors. Despite the achievements, there is still a long way to go in the prevention and treatment of urological tumors.

The microbiome, a term referring to the sum total of all microorganisms living on and within the human body, encompasses not only bacteria but also a wide range of microbial species, such as fungi and viruses (20). There is a delicate and complex balance between these microscopic lifeforms and the human body, and they play a vital role in several ways (21). For maintaining human health, the microbiome is indispensable. It prevents the invasion and colonization of foreign pathogens by occupying ecological niches and producing antimicrobial substances, etc., thus protecting the host from infection (22). In addition, the microbiome aids in nutrient absorption and utilization in the human body. For example, gut microbes can break down indigestible food components like cellulose and produce beneficial substances such as short - chain fatty acids (SCFA), which provide the body with energy and promote the growth and repair of intestinal cells (23). Besides the above functions, the microbiome has a significant impact on the regulation of the immune system. It participates in the development and operation of the human immune system by interacting with immune cells, inducing immune tolerance or activating immune responses. This immunomodulatory effect helps maintain the homeostasis of the immune system and mobilizes immune resources against pathogens during disease onset (24). When the balance of the microbiome is disturbed, this harmonious symbiosis may turn into a pathogenic factor. Microbiome imbalances may contribute to the development and progression of various diseases, including but not limited to intestinal inflammation, metabolic diseases, and autoimmune diseases. The development of these diseases is often closely related to factors such as reduced microbiome diversity, over proliferation or absence of specific microbial species (23, 25–27). However, with the continuous development of detection techniques, tissues or organs previously considered sterile, such as the kidney and prostate, and urine have also been shown to contain small amounts of microbial communities (28, 29). Additionally, tumor tissues have been shown to be present in the tumor microbiota, which is considered part of the tumor microenvironment and plays an important role in tumor development (30). Overall, the microbiome, as an important component of the internal and external environment of the human body, plays an irreplaceable role in maintaining human health, regulating the immune system, and promoting nutrient absorption. An in - depth study of the interactions between the microbiome and the human body will not only help us better understand the mechanisms of the microbiome’s role in health and disease, but also provide new ideas and methods for disease prevention and treatment.

## 2 Microbiome in urinary tumors

In the human body, the gut flora was one of the first microbiomes to draw our attention. As early as reported in1944, the discovery of the gut microbiota (GM) confirmed that the microorganisms in the gut are an integral part of the human body and are closely related to human health (31). With the development of 16S rRNA sequencing, an increasing number of diseases, especially tumor diseases, have been found to be closely related to gut flora. In addition, advances in detection technology have enabled us to study microorganisms outside the gut, such as those in urine and tissues. Similar to the gut flora, these microorganisms located at different sites may be involved in tumorigenesis and development in various ways and may also serve as potential molecular markers for predicting tumor formation. Here, we review the microbiota at various sites associated with urological tumors and describe the pathways by which they are involved in tumor development. A preliminary summary of the role of these microorganisms in urinary tumors is presented.

### 2.1 Prostate cancer

The development of prostate cancer is influenced by various factors, including lifestyle changes, endocrinology, and inflammation. However, as researchers began to consider microorganisms, they realized that alterations in microbial levels were also strongly associated with prostate cancer development. A study by Boursi et al. confirmed a slight increase in the risk of prostate cancer after repeated exposure to antibiotics such as penicillin, quinolones, sulfonamides, and tetracyclines. They concluded that the prolonged use of these antibiotics disrupts the normal microbial structure of the gut, ultimately leading to an increased risk of prostate cancer (32). On the contrary, another study (33) showed that Astragaloside IV co - peptides derived from scorpion venom (PESV) treatment can inhibit prostate cancer tumor growth by restoring the GM and microbial metabolic homeostasis through the AGE - RAGE pathway. Similarly, an *in vivo* study (34) confirmed that fecal microbiota from healthy control mice significantly reduced TNF - α levels and inhibited tumor growth when transplanted into CRPC mice. This indicates that gut microbial homeostasis is important for prostate cancer development. Subsequently, two Mendelian randomization studies (35, 36) evaluated the association between several gut flora and prostate cancer, and the two studies yielded consistent results; specifically, the level of *Alphaproteobacteria* in the gut was negatively associated with the risk of prostate cancer. The above - mentioned studies initially identified gut microorganisms at the phylum level associated with prostate cancer development. Furthermore, a meta - analysis (37), which included 7 research papers with a total of 250 prostate cancer patients and 192 controls, further assessed the gut flora closely associated with prostate cancer at the bacterial phylum level. Their results showed that in terms of gut flora abundance, among prostate cancer patients, the abundances of *Proteobacteria*, *Bacteroidia*, *Clostridia*, *Bacteroidales*, *Clostridiales*, *Prevotellaceae*, *Lachnospiraceae*, *Prevotella*, *Escherichia*, *Shigella*, *Faecalibacterium*, and *Bacteroides* were higher. Conversely, in the control group, the abundances of *Actinobacteria*, *Bacteroidetes*, *Firmicutes*, *Selenomonadales*, *Veillonella*, and *Megasphaera* were higher. In addition, a study (38) that compared the gut flora of patients in the high - risk, low - risk, and control groups by 16S rRNA sequencing found that the levels of several specific gut microbes, namely *Rikenellaceae*, *Alistipes*, and *Lachnospira*, were significantly increased in the high - risk group. This suggests that the GM is not only closely associated with tumorigenesis but may also further influence tumor progression, such as invasion and metastasis.

However, instead of regarding the microbiota as an independent risk factor, we need to focus on its role in the host, specifically, what processes in the host it influences to contribute to tumorigenesis and progression. As previously mentioned, prostate cancer is associated with various factors, including poor lifestyle habits, endocrine metabolism, and inflammation, and we will continue to explore how the gut flora is involved in tumor progression through its participation in these factors. We know that poor dietary habits, such as a high - fat diet, are a major risk factor for prostate cancer, and that long - term intake of high - fat foods, especially those rich in saturated and trans fats, can lead to elevated androgen levels in the body. In addition, a study (39, 40) confirms that a high - fat diet also promotes prostate cancer progression by affecting the abundance of specific gut microbial species as well as the levels of genes involved in lipid metabolism. Altered endocrine levels are another major factor in prostate cancer development, and one study (41) found that through a functional analysis of the differential gut flora associated with prostate cancer, the majority of the GM were associated with steroid biosynthesis (5 - alpha reductase) metabolism. In addition, GM have been shown to activate local MAPK and PI3K pathways via their - derived short - chain fatty acids, which in turn affect IGF1 signaling and ultimately prostate cancer growth (41). Finally, the influence of the GM on host immunoinflammation is also a key factor in promoting tumor progression. Transplantation of feces from CRPC patients into normal control mice revealed an increase in SCFA-producing microorganisms, including *Ruminococcus*, *Alistipes*, and *Phascolarctobacterium*, and a corresponding increase in their intestinal levels of SCFAs. These SCFAs, on the one hand, enhanced PCa cell migration and invasion by inducing TLR3 - triggered autophagy to further activate NF - κB and MAPK signaling, and on the other hand, reprogrammed the tumor microenvironment through enhanced autophagy by releasing higher levels of the chemokine CCL20, which recruited more macrophage infiltration and simultaneously polarized them to the M2 type (42). Similarly, another study demonstrated that *Cutibacterium acnes* in the gut induces the expression of macrophage - immunity genes and can influence the levels of regulatory T cells within the tumor microenvironment (43). In addition, the levels of several pro - and anti - cancer molecules in the host are closely linked to the gut flora. Elevated levels of phenylacetylglutamine (PAGln) greatly increase the likelihood of developing high - risk prostate cancers, and the presence of microorganisms in the gut that catabolize the amino acid phenylalanine to produce PAGln is considered a significant risk for invasive prostate cancers (44, 45).

As the notion that urine is sterile has been refuted, researchers have begun to study the urinary microbiome and have found that, in addition to gut microbiome differences, there are also differences in the microbial composition of urine between prostate cancer patients and healthy individuals. A study by Hurst et al. (46) confirms that urinary microbes are associated with a higher risk of prostate cancer and identifies five prognostic species through bacterial testing of urinary sediment associated with poor prognosis. A systematic review of the urologic microbiome and prostate disease (47) also indicated that specific genera/species such as *Streptococcus*, *Bacteroidetes*, *Faecalibacterium*, *Lactobacilli*, and *Acinetobacter* were found in higher abundance in the urine of prostate cancer patients. In addition, Alanee et al. (48) collected urine from patients with transrectal prostate biopsies for a before - and - after comparison and found that when the prostate biopsy was negative, the level of *Lactobacillus* in the urine was high, whereas when the biopsy was positive, the level of *Lactobacillus* in the urine was reduced and replaced by a higher level of *Prevotella bacteria*. In addition to showing significant differences in the urinary microbiome compared to healthy controls, there were also differences in the urinary microbiomes of patients with different - grade tumors (49), implying that, like the gut flora, urinary microbes may be further involved in tumor progression. A study (50) confirmed that prostate tissue contains a large number of bacteria, mainly located in the gland, and that there is a pathophysiological correlation between its microbial composition and prostate cancer development. This finding provides an interesting perspective for prostate cancer research and treatment. Subsequently, several genomic studies (51, 52) comparing the macrogenomic differences between prostate tumors and adjacent benign tissues have also confirmed the non - sterile environment in the prostate gland and some differences in the microbial composition between prostate cancer tissues and benign tissues, such as a significantly higher abundance of *Shewanella* in tumor tissues and reduced species diversity in *Staphylococcus saprophyticus* and *Vibrio parahaemolyticus*. Similarly, the microbiota in the tumor tissue was strongly associated with the level of tumor progression or prognosis. It was found that high - grade prostate cancer tissues were enriched with more *Cutibacterium*, *Pelomonas*, and *Corynebacterium* genera compared to low - grade prostate cancer tissues. Furthermore, Kim (53, 54) compared the microbiota of biochemically recurrent and recurrence - free prostate cancer tissues and found that the abundance of *Lactobacillus* was relatively high in the recurrence - free group, further emphasizing the role of *Lactobacillus* in prostate defense.

### 2.2 Bladder cancer

Similar to prostate cancer, bladder carcinogenesis is closely related to the GM, despite not being part of the gastrointestinal tissue. A case - control study in China comparing the differences in GM between bladder cancer patients and normal controls found that the abundances of *Clostridium cluster* XI and *Prevotella* were reduced in bladder cancer patients (55). The effect of GM on bladder cancer can be achieved, on the one hand, by modulating the body’s inflammatory immune response. As He et al. found, normalization of the GM using radicicol thiols reduces the inflammatory and immune response, thereby preventing chemically - induced bladder cancer (56). On the other hand, GM metabolites are also important means by which the GM influences tumors in distal tissues. For example, the human GM can influence the toxic metabolism of nitrosamines, and its depletion significantly reduces the progression and severity of nitrosamine - induced bladder cancer (57). Similarly, metabolites of the GM can influence retinoic acid metabolism and thus the preventive effect of microbial A on bladder cancer via various pathways (58).

As a microbiota that can directly contact the bladder, in contrast to the GM, the urine microbiome has been extensively studied in relation to bladder cancer development. Several systematic reviews of the literature (59–61) have summarized the changes in the urine microbiome of bladder cancer patients, and we have also compiled them in **Table 1**. As demonstrated in the table, although there are large discrepancies among the results of these studies, probably due to differences in the characteristics of the study subjects and technical reasons for urine sampling, a more consistent trend can be summarized. For example, the abundances of *Fusobacterium* and *Actinobacillus* were higher in the urine of bladder cancer patients, while, in contrast, the abundances of *Lactobacillus* and *Weyronella* were higher in the urine of the control group. In addition to showing deviations from normal in the bladder cancer patients’ urine microbiome, more interestingly, it has been found that the urine microbiome composition of some patients with non - invasive bladder cancer slowly changes over time after transurethral resection and was even found to regress to previously normal levels in some recurrence - free tumor groups (53, 73). Similarly, the urinary microbiome was also strongly associated with patients’ postoperative survival and recurrence (74). In addition, there was also a difference in the abundance of the urine microbiome among patients with different tumor grades (64). This implies, on the one hand, that the urine microbiome plays an important role in bladder cancer and, on the other hand, suggests its potential as a prognostic marker for bladder cancer treatment. However, despite this, studies related to the urine microbiome have faced major challenges. One of the biggest problems is the collection and contamination of urine samples. Hourigan et al. compared mid - stream urine samples with those obtained by cystoscopy and found that in male patients, there was a significant difference between the urine microbiomes identified by the two urine collection methods (75). Similarly, there have been reports on the microbiota of bladder cancer tissues. Pederzoli ‘s study (62) showed a higher abundance of *Burkholderia* in tumor tissues compared to non - tumor tissues. Another study (72) that compared 22 cancerous tissues and 12 normal tissues adjacent to the cancer found lower relative abundances of *Lactobacillus*, *Prevotella_9*, and *Ruminococcaceae*, while *Cupriavidus*, *Brucellaceae*, *Acinetobacter*, *Anoxybacillus*, *Escherichia*, *Shigella*, *Geobacillus*, *Pelomonas*, *Ralstonia*, and *Sphingomonas* had higher relative abundances in cancer tissues. In addition, it has been shown (76) that microbiota detection in samples from cancerous tissues is highly reproducible and independent of the given bladder tissue collection site when compared to the urinary microbiome. In addition, microbial genera such as *Akkermansia*, *Bacteroides*, *Clostridium sensu stricto*, *Enterobacter* and *Klebsiella* are more representative in bladder cancer identification in tissues compared to the urinary microbiome.

**Table 1:** Overview of urine microbiome in bladder cancer.

Periode	Males/females	Age	Case/control	Pathology	Increase	Decrease	Reference
not mentioned	70/38	not mentioned	49/59	urothelial carcinoma	*Klebsiella*, *Enterobacteriaceae*	not mentioned	(62)
not mentioned	not mentioned	>40	51/10	urothelial carcinoma	*Veillonella*, *Corynebacterium*	*Ruminococcus*	(63)
2017.03-2017-09	49/0	45-69	31/16	urothelial carcinoma	*Acinetobacter*, *Anaerococcus*, *Sphingobacterium*	*Serratia*, *Proteus*, *Roseomonas*	(64)
2017-2019	81/0	35-65	62/19	urothelial carcinoma	not mentioned	*Lactobacillus*	(65)
2015.10-2016.10	23/0	not mentioned	12/11	urothelial carcinoma	*Fusobacterium*, *Actinobaculum*, *Facklamia*, *Campylobacter*, *Subdoligranulum*, *Ruminococcaceae*	*Veillonella*, *Streptococcus*, *Corynebacterium*	(66)
2017.01-2017.03	18/6	30-86	24/0	not mentioned	*Acinetobacter*	not mentioned	(67)
2015.11-2016.01	35/20	28-88	29/26	urothelial carcinoma	*Actinomyces europaeus*	*Streptococcus*, *Bifidobacterium*, *Lactobacillus*, *Veillonella*	(68)
2019.08-2021.02	75/0	44-78	34/29	not mentioned	*Veillonella*, *Varibaculum*, *Methylobacterium*	*Pasteurella, Corynebacterium, Acinetobacterium*	(69)
2018.03-2019.09	44/9	about 70	43/10	urothelial carcinoma	*Actinomyces*, *Achromobacter*, *Brevibacterium*, *Brucella*	*Salinococcus*, *Jeotgalicoccus*, *Escherichia*, *Faecalibacterium*, *Thermus*, *Lactobacillus*	(70)
2018.08-2019.05	not mentioned	about 71	38/10	urothelial carcinoma	*Bacteroides*, *Lachnoclostridium*, *Burkholderiaceae*	*Bacteroides*, *Faecalbacterium*	(71)
not mentioned	34/0	56-75	22/12	urothelial carcinoma	*Brucellaceae*, *Acinetobacter*, *Anoxybacillus*, *Escherichia*, *Geobacillus*, *Pelomonas*, *Ralstonia*, *Sphingomonas*	*Lactobacillus*, *Prevotella_9*, *Ruminococcaceae*	(72)

### 2.3 Renal cancer

Unsurprisingly, the microbiota has also been reported to be associated with the development of renal malignancies. A Mendelian randomization study (77) involving 248,356 benign tumors and 238,678 renal malignancies genetically controlled for intestinal microbiota found that *Actinobacteria*, *Intestinimonas* and *Veillonella* were protective against renal carcinoma, whereas higher levels of intestinal *Enterobacteriaceae* increased the risk of kidney cancer. Another similar study (78) also showed that certain microbial genera in the gut, such as *Alphaproteobacteria*, *Bacilli*, *Coprococcus2*, *Intestinimonas*, *Lachnoclostridium*, *Lactococcus*, *Ruminococcus torques* and *Eubacterium brachy*, have been associated with renal cell carcinoma. Similarly, a study (79) evaluated the differential expression of microbiota in renal cancer tissues. A comparison of 16S rRNA sequencing of tumors and adjacent normal tissues of 24 patients with renal cell carcinoma revealed that some genera, such as *Nitriliruptor*, *Deinococcus*, *Actinomyces*, *Gordonia*, *Pseudoclavibacter*, *Microlunatus*, *Amycolatopsis*, *Weissella*, *Brevundimonas* and *Phyllobacterium*, were abundant in renal cancer tissues; however, other genera, such as *Geothrix*, *Bifidobacterium*, *Paenisporosarcina*, *Alloiococcus*, *Caloramator*, *Allobaculum* and *Pseudoclavibacter*, were also found to be abundant in renal cancer tissues, while *Caloramator*, *Allobaculum*, *Rhodoplanes*, *Carludovica*, *Novosphingobium*, *Dechloromonas*, *Klebsiella*, *Coxiella* and *Pseudomonas* were predominantly in normal tissues. Also, Heidler et al. (80) compared microbiome expression differences in benign and malignant kidney tumor tissues and found that the microbiomes of *Aeromonas salmonicida*, *Pseudoalteromonas haloplanktis*, *Parageobacillus toebii*, *Trachelomonas volvocinopsis*, *Mycoplasma mycoides* and *Halomicrobium mukohataei* are particularly common in cancer tissues.

It can be seen that microbiota colonizing the gut, urine, and tissues are closely associated with the occurrence, development, and treatment of urinary tumors. This indicates the important role of the gut microbiota in urinary tumors. In recent years, a great deal of research has been conducted on the mechanisms between microbiota and urinary tumors, and the involvement of microbiota in the regulation of tumor therapy has been continuously explored. However, summaries regarding the microbiome and immunotherapy of urinary tumors are scarce. Several recent studies have found that antibiotic intervention can influence tumor immunotherapy in certain tumors, which is thought to be associated with gut flora dysbiosis. However, these studies are only at a preliminary stage, and as summarized earlier, a considerable number of microbiota species located at different sites are strongly associated with urological tumors and are involved in tumor development through various pathways. Therefore, it is necessary to comprehensively compile these elements.

## 3 Role of microbiome in urinary tumor immunity

In the last two decades, modulation of the tumor immune response has emerged as a reliable option for tumor therapy. Similarly, as gene sequencing technology continues to evolve, there is growing evidence that the composition of the microbiota (both intestinal and extraintestinal) confers susceptibility to certain tumor diseases, as well as influencing cancer outcomes, and it has been demonstrated that this correlates with modulation of the immune response (81, 82). A large body of data (83–88) suggests that testing the microbiota of oncology patients can be used as a marker to predict a patient’s response to chemotherapy or immunotherapy, as well as to predict treatment-related toxicity. This means that shaping the microbiota is expected to be a new target for cancer treatment efficacy and toxicity. This exciting result has attracted the interest of many researchers, who have begun to employ a variety of strategies to shape the microbiota to enhance anticancer efficacy. Several preliminary trials (85, 87–89) have shown that fecal micro transplantation (FMT), in combination with immunotherapy, induces differential expression of T-cell and NK-cell related pathways to control tumors and improve the immune response; similarly, the use of probiotics (*Lactobacillus* and *Bifidobacterium*), whose substrates are selectively utilized by the host microorganisms to confer health benefits, can also promote immune modulation, among other things, to alleviate dysbiosis and enhance anticancer efficacy and immunity and immune checkpoint inhibitor (ICI) therapeutic effects (84, 90, 91). In addition, with the development of gene technology, some engineered microbial products have attracted the attention of a large number of researchers due to their unique advantages. To date, several research reports have confirmed that a variety of engineered bacterial strains reliably provide antitumor benefits in many different settings (92–94). However, despite the rapid development of the concept of the gut microbiota as a precision medicine tool over the past decade, the number of published studies on practical interventions to modify the gut microbiota is relatively limited and lacks specificity The promise of the gut microbiome as part of individualized treatment strategies (95). Further elucidation of the microbiota and tumor immunity mechanisms will help drive its further development.

As is known, immunotherapy comprises active immunotherapy and passive immunotherapy. In urologic tumors, the former mainly involves bladder BCG infusion, while the latter mainly includes monoclonal antibodies, such as some immunocheckpoint inhibitor drugs like PD - 1/PD - L1 and CTLA - 4 antibodies. Significant efficacy has been achieved in many malignant tumors, including urologic tumors (96–98). It is worth noting that there is growing evidence that these groups of organisms, to which we have previously paid little attention, play an important role in the use of these drugs for the treatment of urologic tumors. Knorr et al. classified patients with high - risk non - muscle - invasive bladder cancer as BCG non - responsive and BCG - responsive (defined as no evidence of tumor recurrence within 2 years of BCG treatment) and found that the *Lactobacillus* was significantly enriched in BCG - responders (99). The use of tyrosinase inhibitors and antibiotics prior to the use of nivolumab for advanced renal cell carcinoma significantly affects the treatment efficacy, which is thought to be closely related to the fact that tyrosinase inhibitor and antibiotic use affects the microbiological composition of patients (84). Similarly, another study has confirmed significant changes in the fecal microbial composition during the treatment of patients with metastatic renal cell carcinoma with PD - 1. Furthermore, this study found that patients with higher microbiota diversity tended to have a better response to treatment. All of this suggests that the composition of some specific microorganisms is closely related to tumor immunotherapy. However, this is insufficient to address the challenges of tumor immunotherapy in the clinic. Further exploration of various types of microbiota in tumor immunity and the molecular mechanisms of immunotherapy is the key to solving this problem.

A number of “checkpoint” mechanisms exist in the human immune system for regulating the strength of the immune response. Among them, cytotoxic T - lymphocyte - associated antigen 4 (CTLA - 4), PD - 1, and PD - L1 are the most intensively researched immune checkpoints. PD - L1 is a molecule expressed on the tumor cell membrane surface, while its receptor, PD - 1, is expressed on the T - cell surface. The binding of PD - L1 to PD - 1 negatively inhibits signaling initiation and ultimately inhibits T - cell activation, proliferation, and cytokine secretion (e.g., IFN - γ, IL - 2, etc.) (100). Since 2015, ICIs have been successively approved by the U.S. Food and Drug Administration (FDA) for the treatment of renal and uroepithelial cancers (101, 102). In addition, although many studies (103–105) have shown that ICIs exhibit limited therapeutic activity in prostate cancer, there is also evidence indicating some therapeutic promise of ICIs in prostate cancer (106). As we mentioned earlier, the balance of the microbiota plays an important role in host physiological functions, especially in shaping the body’s normal and abnormal immune responses, which in turn affects tumor promotion or suppression. This suggests that the role of the microbiota in antitumor immunosurveillance cannot be underestimated and could be a potential biomarker of response to ICIs. As hypothesized, the microbiota we focused on proved to be a key factor that may be partially responsible for the lack of response to treatment with ICIs. Evidence from preclinical mouse models suggests that *Alistipes* gavage restores the response of TNF-dependent tumors to immunotherapy, whereas *Lactobacillus* inhibit the response (107). Similarly, Sivan et al. (108) found that mice carrying different microbiota presented different results in terms of anti-tumor immunity and melanoma growth. Primarily, the abundance of *Bifidobacterium* was shown to correlate with T-cell responses, and anti-PD - L1 treatment combined with transoral feeding of these bacteria nearly eliminated tumor growth by increasing dendritic cell maturation and anti-tumor CD8+ T-cell activity. Evidence from multiple clinical patients has led to similar conclusions. In treated patients with metastatic melanoma, a variety of bacteria (*Faecalibacterium prauznitzii*, *Holdemania filiformis* and *Dorea formicigenerans*) were associated with anti-CTLA - 4 plus anti-PD - 1 clinical efficacy (85, 109). In patients with non-small cell lung cancer (NSCLC) or uroepithelial carcinoma treated with anti-PD - 1, baseline microbiota analyses showed that patients with clinical benefit had higher microbiota richness. A higher distribution of the *Firmicutes*, as well as *Akkermansia* and *Alistipes*, was found in responding patients (88). However, some patients with certain tumors exhibit primary resistance to ICIs. Moreover, even in some tumors where ICIs are generally beneficial, patients show large variations in treatment outcomes (96, 97, 110). Therefore, we need to further clarify the reasons for this and the composition of microorganisms associated with urologic tumors to provide a good starting point.

The Bacillus Calmette - Guérin (BCG) vaccine is an important means for preventing tumor recurrence in non - muscle - invasive bladder cancer (NMIBC). Additionally, BCG can also delay tumor progression and reduce the risk of tumor infiltration into the muscularis propria and even distant metastasis, and the therapeutic efficacy of BCG is more pronounced in high - risk NMIBC patients (111, 112). A recent study (113) evaluated the accuracy of the NMIBC scoring model in predicting disease progression in patients with high-risk stage T1 NMIBC treated with BCG. The study demonstrated that more than 70% of patients did not progress to muscle-invasive disease after intravesical BCG infusion following re-TURB, confirming the benefit of BCG therapy in these patients. Although the therapeutic mechanism of BCG is still unclear, available evidence suggests that innate and adaptive immunity play key roles in BCG treatment, which produces pathogen-associated molecular patterns that can be recognized by host pattern recognition receptors (e.g., TLR2, TLR4, and TLR9, etc.), which induces MyD88 signaling to regulate pro-inflammatory cytokine production, and MyD88 deficiency leads to reduced response to BCG treatment in mice (114). In the urine and bladder wall of BCG-treated patients, the number and activity of several innate immune cells, including macrophages (115), polymorphonuclear cells (116), dendritic cells (117), and natural killer cells (118), were altered, and removal of these cells abrogated the efficacy of BCG treatment in the mouse model. In terms of adaptive immunity, T cells are central to the treatment of bladder cancer with BCG, and their presence can be detected in the urine and bladder mucosa of patients treated with BCG for several months (119). In addition, BCG shifts the urinary cytokine milieu from Th2 to Th1 (120, 121), and changes in the levels of Th1-associated cytokines (e.g., IL-12 and interferon γ, etc.) are strongly correlated with the clinical response. The mechanisms by which BCG is used in the treatment of bladder cancer are multifaceted and interrelated. During the interaction with uroepithelial cells, BCG adheres to them, and the likely mechanism is that Mycobacterial fibronectin adhesion protein binds to host fibronectin, which in turn attaches to uroepithelial cells (122). Bladder cancer cells can uptake BCG, and after uptake they secrete a variety of immune-activating effectors, which may recruit or activate immune cells, although there is no definitive *in vivo* evidence that this uptake process is necessary for the efficacy of BCG (123). At the same time, BCG has a direct toxic effect on bladder cancer cells, which has been demonstrated *in vitro* in experiments with high ratios of BCG to cancer cells, but the *in vivo* situation is not clear (124, 125). Encouraging data show that BCG has been effective for more than 40 years, significantly reducing long-term tumor recurrence, risk of progression and mortality. A Southwest Oncology Group randomized study (126) showed a 5-year survival rate of 78% in the no BCG maintenance group compared to 83% in the maintenance group, suggesting that BCG immunotherapy is beneficial for patients with carcinoma *in situ*, as well as for selected patients with Ta and T1 bladder cancers. Similarly, Herr (127) evaluated 86 patients and found that the mortality rate decreased from 32% to 14% with BCG. A subsequent follow-up report (128) noted that patients who adhered to BCG therapy experienced a reduction in cancer mortality from 37% to 12% within three years. Despite the low number of serious side effects of BCG, some common adverse reactions such as urinary frequency, urgency, nocturia, bladder pain, low-grade fever, chills, and hematuria have been reported and most of the symptoms subside within 48 hours of BCG instillation (129). In addition, some rare adverse events have been reported, including Reiters syndrome (130), parotid infection (131), arteriovenous dermatocutaneous fistulae (132), psoas major muscle abscess, rupture of the iliac artery (133), and Poncet’s disease (134). Moreover, there are still some limitations of current BCG therapy. For example, the following adverse side effects have been reported during treatment (135). Secondly, in some immunocompromised individuals, such as the elderly, the therapeutic efficacy is greatly reduced (136), and tumor recurrence has been reported in about 30% of patients within the first 3 years of BCG treatment (137). Similarly, the microbiome has become a key target for addressing these issues, especially in urine, as it can come into close contact with BCG and tissues.

### 3.1 Role of gut microbiota in immunity to urologic tumors

When considering the influence of gut flora on tumor immunomodulation, especially in non - gastrointestinal tumors, the first question to consider is by what means the gut flora transmits regulatory information. The next step is to explore the tumor - immune - related pathways in which the gut flora is involved. A study on the microbial mechanisms of pancreatic ductal carcinoma (138) detected microorganisms within the tumor, and it was hypothesized that they entered and elicited a cancer response via retrograde migration from the duodenum to the pancreatic duct. This suggests that intestinal microorganisms can induce carcinogenesis through direct invasive effects on tissues. However, this explanation is not applicable to urinary tract tumors because, under normal conditions, microorganisms from the gastrointestinal tract are virtually incapable of directly invading the urinary tract. Some studies (139, 140) have summarized the pathophysiological links between the gut microbiota, intestinal permeability, and the genitourinary tract, providing a referable basis for our speculations, in addition to directly inducing carcinogenesis, host immune factors and immune cells are considered effective “messengers” for intestinal microorganisms. They can spread throughout the body by driving immune recognition in the intestinal epithelium or mesenteric lymph nodes and inducing immunomodulatory factors to enter the circulation. Subsequently, circulating immune factors and immune cells can transmit signals from the intestinal microbes to the tumor - immune microenvironment or tumor - draining lymph nodes (141). Finally, microbial metabolites are also among the factors that enable the gut flora to influence a wide range of pathophysiological processes in the host. They can also enter the blood circulation and be transported throughout the body, thereby not only regulating host metabolism (142) but also influencing the activity and function of various immune cells such as macrophages and B cells (143). Therefore, we began exploring the mechanism of immunomodulation of urological tumors by GM from these “messengers”.

Wang et al. (144) examined the efficacy of PD - 1 immunotherapy in MB49 tumor - bearing mice by performing FMT with *Parabacteroides distasonis*. It was found that the introduction of *Parabacteroides distasonis* enhanced the efficacy of PD - 1 immunotherapy, as evidenced by delayed tumor growth and increased densities of CD4+ T and CD8+ T cells in the tumor. In addition, transcriptome analysis further revealed that *Parabacteroides distasonis* supplementation by gavage further enhanced the levels of multiple anti - tumor immune pathways, including natural killer cell - mediated cytotoxicity, T - cell receptor signaling pathway, B - cell receptor signaling pathway, and chemokine signaling pathway. This study provides preliminary evidence that the GM modulates the immune response to urologic tumors through immunokines and immune cells acting as “messengers”. Another study (88) analyzed the immunological changes induced in mesenteric lymph nodes (mLN), tumor - draining lymph nodes (dLN), and tumor tissues after oral gavage of *Akkermansia muciniphila* and *Enterococcus hirae*. It was found that central memory (TCM) CD4+ T cells expressing the chemokine receptor CXCR3 were enriched in the mLN 48 h after injection. Subsequently, these CD4+ T cells were observed in the dLNs and tissues after being killed, respectively. This implies that the GM achieves its immune - killing effect on tumors by inducing immune cell differentiation in the mesenteric lymph nodes and reaching the tumor site through the circulation. It is worth noting that this study also found that specific gut microbes were strongly associated with tumor immunotherapeutic efficacy by modulating specific peripheral T - cell subset responses (e.g., Th1 and Tc1 cell responses against *Akkermansia muciniphila* and Tc1 responses against *Enterococcus hirae*) in the context of PD - 1 blockade therapy. This result once again suggests that there are “key members” of the GM that regulate the immune response to urologic tumors through specific signaling molecules.

**Figure 1:**
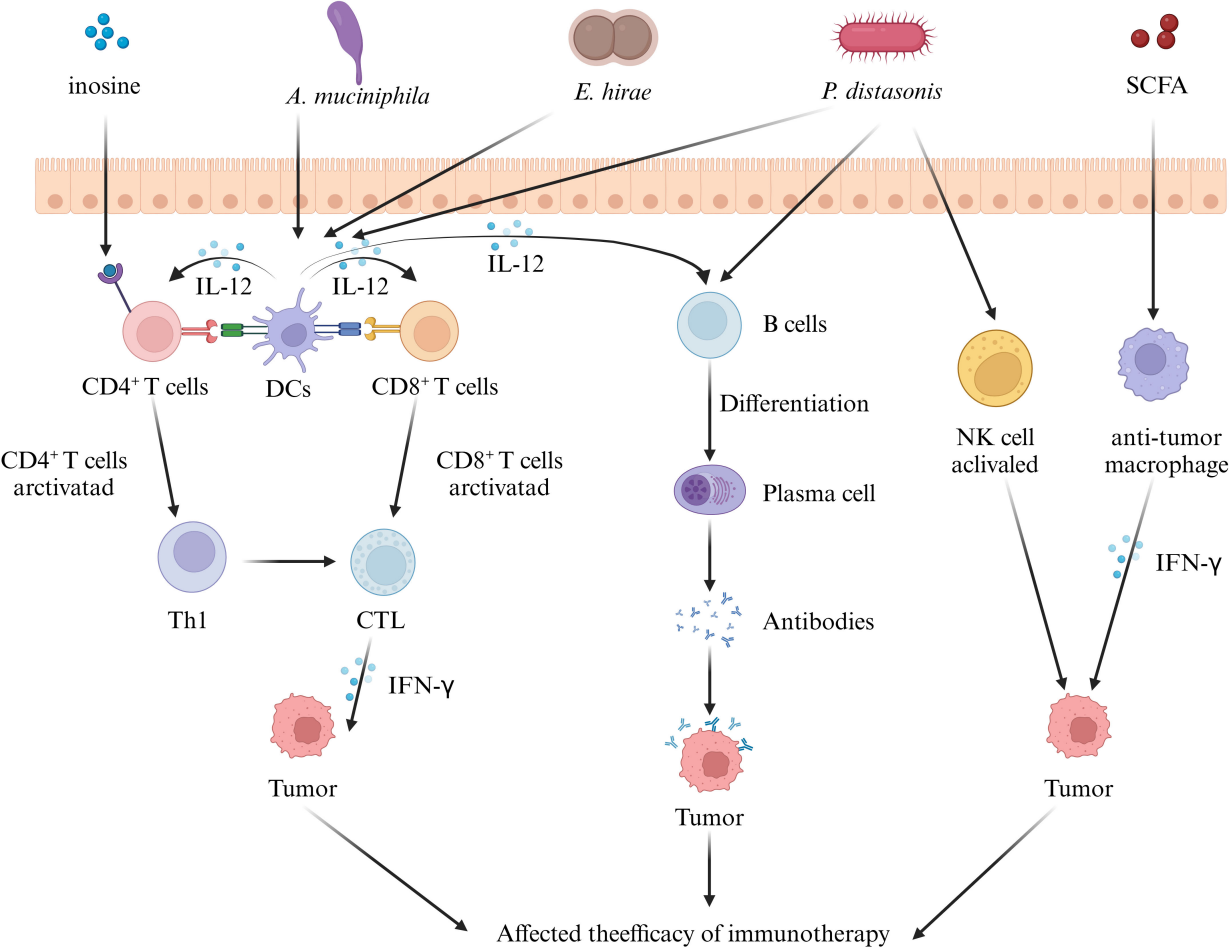
Effect of gut microbiota on tumor immunity in urologic tumors. Some intestinal microorganisms induce the differentiation of immune cells in mesenteric lymph nodes. These immune cells and cytokines reach urological tumor tissues via the circulation and influence tumor immunity by regulating the T - cell, B - cell, and NK - cell signaling pathways. In addition, the metabolites of the gut microbiota can also act as messengers, reach the tumor tissues, and regulate the immune response, ultimately influencing tumor immunotherapy. (IL, interleukin; SCFA, short chain fatty acid; DC, dendritic cell; CTL, cytotoxic t lymphocyte; IFN-γ, interferon-γ).

We next focus on the role of another “messenger” of the gut flora in urologic tumor immunity. Liu ‘s *in vivo* and ex vivo studies (42) have amply demonstrated that GM - derived SCFA, as mediators linking the GM to prostate cancer, are closely associated with prostate cancer progression. On the one hand, their *in vivo* study found that colonizing the intestinal tract of mice with fecal suspensions from CRPC patients resulted in an increase in the number of SCFA - producing flora and a corresponding increase in the levels of SCFAs such as intestinal acetate and butyrate. On the other hand, *in vitro* experiments showed that SCFAs induced Toll - like receptor 3 (TLR3) activation and further induced autophagy activation in prostate cancer cells. Autophagy activation was further accompanied by NF - κB and MAPK signaling, ultimately regulating the invasion and immune - inflammatory response of prostate cancer cells. In addition, it was found that the increased level of autophagy resulted in the release of more chemokine CCL20 from prostate cancer cells, which could recruit more macrophages for infiltration and induce their polarization to M2 type for the regulation of the tumor immune microenvironment. Similarly, another study on SCFA and prostate carcinogenesis (145) noted that high - fat diet and antibiotic application affect SCFAs by interfering with the GM, and SCFAs can stimulate the level of insulin - like growth factor - 1 (IGF1) to promote the proliferation of prostate cancer cells. However, this study focused only on the proliferative effects of IGF1 on prostate cells. Given the current evidence that IGF1 interacts with M2 - like macrophages and autophagy (M2 - like tumour - associated macrophage - secreted IGF promotes thyroid cancer stemness and metastasis by activating the PI3K/AKT/mTOR pathway), it is reasonable to speculate that the IGF1 pathway regulated by SCFAs may also have a potential impact on prostate cancer tumor immunity. However, further experiments are required to directly confirm this. In addition, inosine, another metabolite of GM - derived SCFAs, has also been shown to be closely related to the tumor immune response. Roje ‘s study (57) found that serum from mice treated with anti - CTLA - 4 and colonized with *Bifidobacterium pseudolongum* inhibited tumor growth and induced strong anti - tumor immunity in the tumors and spleens of mice. Further metabolomics of the serum samples revealed that the purine metabolite inosine was the only metabolite that was significantly more abundant in the sera of *Bifidobacterium pseudolongum* monocolonized mice. It is worth noting that this study (57) also found that the effect of inosine on T cells requires sufficient co - stimulation (possibly through IL - 12 or IFN - γ production) to achieve efficient anti - tumor immunity. Specifically, in the presence of IFN - γ, inosine strongly promotes TH1 differentiation, whereas in the absence of IFN - γ, inosine exhibits an inhibitory effect on TH1 differentiation. This implies that the activation of GM and exogenous immune factors need to be considered to achieve better tumor immunotherapy. Additionally, this study found that in addition to mediating T - cell effects, inosine may also directly affect tumor cell survival by altering susceptibility to T - cell - mediated killing through direct action on tumor cells (**Figure 1**).

### 3.2 Urinary microbiome in tumor immunity

Unlike the GM, microorganisms in urine are in direct contact with urinary tract tissues. Consequently, the role of the urinary microbiome in tumor immunomodulation centers on the microbial action pathways. Evidence exists that some urinary microorganisms, such as *Fusobacterium*, *Sphingobacterium* and *Enterococcus*, may induce bladder cancer through schistosomiasis (146). In addition, a study by Shrestha et al. (49) found that some pro - inflammatory microorganisms associated with the urinary tract were enriched in the urine of prostate cancer patients. They suggested that these microorganisms may enter the prostate through the biotubes, causing chronic inflammation and atrophy. In addition, a specific type of prostate inflammation, granulomatous prostatitis (GP), has been shown to be closely related to urinary microorganisms (147). Specifically, damage to the epithelium and leakage of prostate secretions leads to reflux of bacterial products in the interstitium, which in turn triggers a strong foreign body inflammatory response in the prostate (148). In addition, BCG-induced GP has been reported in up to 75% of patients treated with BCG, due to reflux of BCG-contaminated urine from the bladder into the prostate (148). Although currently there is no evidence to support the notion that chronic inflammation of the prostate will eventually lead to cancer, it is also regarded as a suspected risk factor for prostate cancer development. We know that prolonged inflammation of mucosal epithelium significantly increases the likelihood of epithelial carcinogenesis, including the urinary tract epithelium. Repeated urinary tract infections can lead to chronic inflammation of the urinary mucosa, resulting in the release of inflammatory factors and reactive oxygen species, which can lead to carcinogenesis. Urinary tract infections have been shown to be closely linked to genitourinary tract microorganisms as well as intestinal microorganisms. Antibiotic therapy may disrupt the ecological balance of urinary microorganisms, exacerbating inflammation and leading to recurrence (149, 150). In addition, ecological imbalances in the gut flora, such as a decrease in SCFAs-producing bacteria, can disrupt the integrity of the intestinal barrier, triggering the “leaky gut syndrome,” which results in the displacement of bacteria and microbial-derived products (MDPs). Upon entering the circulatory system, these substances are able to activate innate immune cells, especially macrophages, causing them to produce pro-inflammatory cytokines, triggering localized inflammation and oxidative stress, creating a chronic inflammatory state (151). At the same time, persistent antigenic stimulation might cause functional depletion of innate immune cells and weaken local immune defenses, thus promoting recurrent urinary tract infections (152, 153). For example, some studies have found that female patients with recurrent urinary tract infections have reduced gut microbial abundance, with a significant reduction in flora associated with propionic acid and butyric acid production, and are associated with low levels of inflammation and specific immune states (154). In contrast, another hypothesis posits that the urinary microbiome may invade extracellular mechanisms that promote or inhibit bladder cancer development (155). We know that the extracellular mechanisms of tumors play a non - negligible role in tumor cell proliferation, invasion, and tumor immunity (156–159). Consequently, we first focus on the effect of urinary microbes on the extracellular matrix of urological tumors (primarily bladder cancer in this case). Under normal conditions, the mucosal barrier on the bladder surface restricts the direct contact between microorganisms in the urine and the extracellular matrix (160). However, when there is persistent bacterial translocation or tissue invasion, for example, when pathogenic bacteria invade and break the mucosal barrier, microorganisms in the urine can come into contact with the bladder tissue or even directly enter the extracellular matrix.

**Figure 2:**
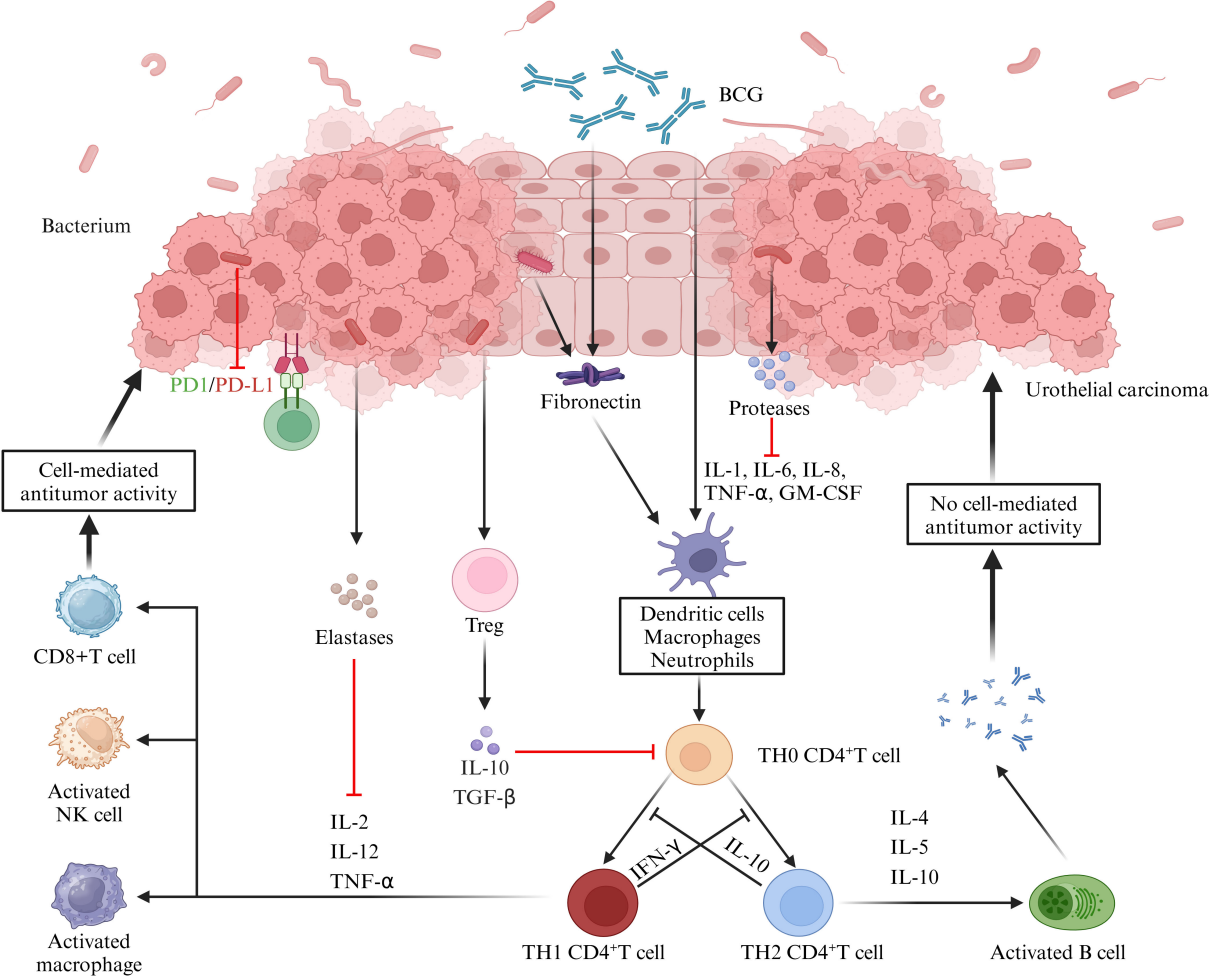
Role of the urine microbiome in the immunotherapy of uroepithelial carcinoma. BCG attaches to the uroepithelium and induces the release of cytokines and chemokines, thereby exerting anti - tumor immunity through the activation of cellular or humoral immunity. Microorganisms in the urine can interfere with these responses. Firstly, bacteria produce proteases or elastases that can directly act on cytokines or degrade cytokine receptors, thereby disrupting the regulation of immune factors. Secondly, some microorganisms can inhibit cell - mediated tumor killing by promoting the infiltration of Treg cells. Additionally, the cross - linking of microorganisms to collagen can further contribute to the therapeutic efficacy of BCG, while other microorganisms can directly inhibit PD - L1 expression, which is important for the immunotherapeutic effect of bladder cancer. (IL, interleukin; BCG, Bacillus Calmette-Guérin; TNF-α, tumor necrosis factor-α; GM-CSF, granulocyte-macrophage colony-stimulating factor; TGF-β, transforming growth factor-β; IFN-γ, interferon-γ).

Specifically, microorganisms directly “invade” tissues by releasing a range of enzymes acting as extracellular virulence factors, which disrupt the host’s physical barriers and immune defense mechanisms (161). For example, some bacteria produce proteases that can disrupt the host’s cytokine regulation by directly acting on cytokines or degrading cytokine receptors (162, 163). These signaling factors include IL - 6 receptors, FAS ligands, and TNF and its receptors (164, 165). In addition, some bacteria release elastases that cleave not only cytokines and receptors but also factors and receptors of the complement system (166). For example, elastases from *Pseudomonas aeruginosa* can modulate the host immune response by inhibiting lymphocyte proliferation through IL - 2 inhibition and IFN - γ inactivation. Urinary microbes have also been shown to potentially influence tumor immunity by acting on T cells. A study on the urinary microbiota for prognostic prediction in non - muscle - invasive bladder cancer (74) demonstrated significant differences in the urinary microbiota between the recurrent and non - recurrent groups. The recurrent group exhibited an enrichment of *Pseudomonas*, *Staphylococcus*, *Corynebacterium* and *Acinetobacter*, and the tumor stroma showed increased infiltration of FoxP3+ regulatory T cells (FoxP3+ Treg), which has been shown to promote immune escape and tumor growth through various mechanisms (167–170). Further correlation analyses showed that the degree of FoxP3+ Treg infiltration was correlated with the urinary microbiology index, albeit not statistically significant, which was mainly attributed to the small number of samples tested. In addition, we further summarize the immune pathways related to the role of urinary microbes in the immunotherapy of urological tumors. An investigative study on urinary microbiology and bladder cancer (70) found that differences in the urinary microbiome were demonstrated not only in patients with non - neoplastic tumors and those with and without muscular infiltration but also in patients who responded and did not respond to BCG therapy. For example, *Serratia* and *Brochothrix* were present in patients who responded to BCG, and *Negativicoccus*, *Escherichia*, *Shigella* and *Pseudomonas* were significantly increased in patients who responded to BCG. Mechanistically, BCG has been shown to modulate tumor immunity by binding to fibronectin, thereby inducing CD8+ T and natural killer cells (171). In contrast, some uropathogens, such as *Lactobacillus casei*, that also bind fibronectin may influence the BCG therapeutic efficacy by enhancing fibronectin stimulation or competing with BCG for fibronectin binding. Another more direct evidence comes from a study investigating the urogenital flora of PD - L1 - positive and negative subjects (172). This study showed higher species richness in the PD - L1 - positive group, which increased with increasing PD - L1 positivity intensity. Meanwhile, the PD - L1 - positive group also showed an enrichment of some bacterial genera (e.g., *Leptotrichia*, *Roseomonas* and *Propionibacterium*) and a decrease in others (e.g., *Prevotella* and *Massilia*). The implication is that these bacteria not only play a key role in tumor immune escape and metastasis but may also serve as potential cofactors to enhance tumor immunotherapy (**Figure 2**).

### 3.3 Tissue/tumor microbiome

Research on tissue microbiota is still in its early stages, and much confusion persists. Firstly, the concept of tissue microbiome is being questioned. Although it has been shown that a variety of microbial DNAs can be detected in prostate cancer tissues (50, 173, 174), there are no direct studies reporting the identification of bacteria through visualization or localization in the corresponding tissue samples. In addition, since the presence of microorganisms is often considered pathogenic, even if microorganisms are present in the tissues, are these microorganisms, like those in our intestines, in a mutually beneficial symbiotic relationship with the host rather than having a pathogenic infectious role? Therefore, in the absence of clear direct evidence, we believe it is inappropriate to use the concept of tissue microbiome to summarize its relationship with immunity to urological tumors. Therefore, we directly focused our attention on intra - tumor microbes and concentrated on the relevant immunomodulatory role of tumor microbes in urologic tumors. It has been demonstrated (175, 176) that certain microorganisms may be present in the tumor itself as part of the tumor microenvironment, and they may be of circulating origin or from normal adjacent tissues. They are not randomly present in the tumor tissue; instead, they are specifically present and highly organized within tumor cells, epithelial cells, and immune cells (175, 177). In addition, it should be noted that although current evidence cannot indicate whether these microbes in tumors play a causal role in cancer development or serve as potential biomarkers for tumor presence (178, 179), they have been noted to be strongly associated with the tumor immune response and immunotherapy efficacy (176). However, this finding has been confirmed in urologic tumors, which are of interest to us. A study by Davidsson et al. (43) found higher Tregs infiltration in *Cutibacterium acnes* - positive prostate cancer tissues. Additionally, their *in vitro* findings showed that *Cutibacterium acnes* stimulation significantly increased the expression of PD - L1 and the chemokines CCL17 and CCL18, confirming the important role of microorganisms in tissues in the tumor immune microenvironment.

## 4 Conclusion and prospect

The microbiome in the human body, an integral part, is closely related to the development of urological tumors. Specific microorganism species may promote tumor cell proliferation and invasion by metabolizing carcinogens, inducing chronic inflammation, or interfering with cell signaling. Differences in microbiota characteristics are manifested not only between tumor and non - tumor patients but also among patients at different tumor stages. Studying these differentially expressed microorganisms is of great significance as they may serve as potential markers for tumorigenesis and progression and as important targets for tumor therapy. In addition, we note that microorganisms from various sites have an important impact on tumor immunity and tumor therapy in urologic tumors. They can enhance the immune system’s killing effect on tumor cells through their metabolites, the production of some enzymes, or their direct action by activating immune cells, inducing tumor cell apoptosis, or changing the tumor microenvironment. These roles of the microbiome and tumor immunity not only well explain the individual differences in the effectiveness of tumor immunotherapy but also, based on these findings, are expected to enable us to develop more efficient and safer microbial immunotherapy regimens, which will bring better therapeutic effects to urological tumor patients. It is worth noting that, as Roje ‘s study (57) pointed out, the effect of inosine, a gut microbe metabolite, on T cells requires sufficient co - stimulation to achieve potent anti - tumor immunity. Thus, in future studies, we cannot simply consider microbes as a single factor, and more interactions need to be further explored. In addition, microorganisms at different sites do not act independently and complementarily, and there are also interactions among them. In future studies, we need to further deepen our understanding of the role of the microbiome in urologic tumorigenesis and development. This includes further elucidating the interactions between microbes and tumor cells and the specific mechanisms by which microbes influence the tumor microenvironment and immune responses. By exploring these mechanisms, we expect to provide new ideas and approaches for treating urologic tumors.

Some significant achievements have been made in microbial modulation approaches for tumor treatment. For example, several studies in pre - clinical trials have shown that microbiota - centered interventions significantly improve the therapeutic outcomes of ICIs (180, 181). In the future, further developing microbial - based tumor vaccines and immunotherapy protocols is also an important research direction. We can utilize known microbial strains with antitumor activity or modify microbes through genetic engineering methods to more effectively stimulate the body’s antitumor immune response. In addition, with the rapid development of big data technologies in software engineering, we can use these technologies to optimize microbiome regulation strategies. For example, by constructing a microbiome database of urological tumor patients and performing algorithmic analysis, we can identify microbial markers closely related to tumorigenesis and development. This not only aids in predicting patient prognosis but also provides a basis for developing individualized treatment plans. Meanwhile, with the help of these technologies, we can also monitor real - time changes in the patient’s microbiome and adjust the treatment plan promptly to achieve the goal of precision medicine. In response to the previously mentioned limitations of microbial detection in tumor tissues, a series of in-tumor microbial detection technological tools have emerged in recent years to overcome these challenges. In addition to the commonly used 16S rRNA gene sequencing, whole metagenome-based shotgun sequencing (WMS) can determine the gene expression status of microorganisms in tumor tissues. By understanding the transcriptional activity of microorganisms, their functional status in the tissue can be inferred (182, 183). In addition, some high-resolution visualization techniques, such as fluorescence *in situ* hybridization (FISH) and immunofluorescence and immunohistochemistry, can be used to directly label microorganisms at the cellular or tissue level, so as to more accurately observe the distribution and localization of microorganisms in tumor tissues (184). It is even possible to construct images of microbial distribution in cancerous tissues in three dimensions, which can be used to explore the interrelationships between microbes and tumor cells as well as the surrounding stromal cells, and to determine whether the microbes are at a possible symbiotic interface or pathogenic invasion site (185). We firmly believe that with the development of technology, tissue microbes will also play a key role in tumor prediction and intervention, just like gut and urine microbes. Future research on the microbiome in urological tumors will focus on exploring the mechanisms of action, developing novel therapeutic regimens, strengthening multidisciplinary cooperation, and optimizing therapeutic strategies with advanced technologies. Through these efforts, we hope to provide more effective treatments for urologic tumor patients and improve their quality of life.
